# Is propofol injection pain really important to patients?

**DOI:** 10.1186/s12871-017-0321-7

**Published:** 2017-02-17

**Authors:** Wen Wang, Linxin Wu, Chaobin Zhang, Li Sun

**Affiliations:** Department of Anesthesiology, Cancer Hospital, Chinese Academy of Medical Sciences, Peking Union Medical College, No. 17 Panjiayuannanli, Chaoyang District, Beijing, 100021 China

**Keywords:** Propofol, Injection pain, Patients’ preferences

## Abstract

**Background:**

Propofol injection pain (PIP) has been adequately studied during the past decades. However, patients’ opinion on this problem and the incidence of patients’ recall of this brief discomfort are still unknown. Thus, we conducted this study to know the patients’ perspectives on PIP and provide useful information about the incidence of recall of PIP under our routine general anesthesia.

**Methods:**

Five hundred preoperative questionnaires were distributed to patients who were scheduled for elective open thyroidectomy under general anesthesia from May 2016 to July 2016. They were asked to rank ten possible adverse effects associated with general anesthesia from their most undesirable to their least undesirable effect. Patients who completed the preoperative questionnaires were asked whether they could recall PIP and to grade the severity of PIP on the first postoperative day.

**Results:**

A total of 448 preoperative questionnaires were returned and analyzed with an efficient rate of 89.6%. Incisional pain was ranked as most undesirable, followed (in order) by vomiting, gagging on the tracheal tube, nausea, sore throat, propofol injection pain, shivering, intravenous puncture pain, and anxiety. The majority (91.5%) of surveyed patients could not recall any discomfort or pain during anesthetics injection. Of those who could recall PIP, 89.5% grade it as mild pain, 7.9% moderate pain, and 2.6% severe pain.

**Conclusions:**

Most of patients undergoing elective open thyroidectomy in our hospital viewed PIP as a relatively minor problem. The incidence of recall of PIP was low and the majority of those who recalled regarded it as mild, temporary and acceptable pain. However, further investigations into propofol injection pain may be warranted as patients’ perspectives on propofol injection pain and its severity may differ between patient populations.

## Background

With the development and improvement of surgical and anesthetic techniques, critical incidents such as cardiac arrest or death during the perioperative period have been obviously minimized. In turn, more attempts have been made to address minor but potentially distressing clinical anesthetic problems such as pain, postoperative nausea and vomiting to further improve the quality of anesthesia care [1].

Propofol injection pain (PIP) is one of these problems and it was ranked seventh among the most important 33 low-morbidity clinical anesthesia problems by a panel of expert anesthesiologists [2]. The issue of PIP has been studied for decades and there are a lot of new clinical studies focused on finding effective interventions every year. However, what is the patients’ opinion on this problem? Is PIP really important to them? Although several studies assessed patients’ preferences for avoiding low-morbidity but common anesthesia problems [3–8], PIP has still not been evaluated. Besides, the incidence of recall of this brief discomfort is still unknown.

Thus, we performed this study to (1) know the patients’ perspectives on PIP by ranking their preferences for avoiding specific low-morbidity but common clinical anesthesia problems and (2) provide useful information about the incidence of recall of this brief discomfort under our routine general anesthesia.

## Methods

### Clinical protocol

A list of the most common but low-morbidity clinical events associated with general anesthesia was derived from previous relevant studies [3–8]. After reviewed by four senior board-certified anesthesiologists in our hospital, nine events including anxiety, intravenous puncture pain, propofol injection pain, gagging on endotracheal tube, shivering, sore throat, nausea, vomiting, incisional pain and a tenth item named as ‘normal’ (without any above event) were chosen for our pilot preoperative questionnaire. Besides, they also edited simple descriptions for each item to facilitate understanding for accuracy. The pilot preoperative questionnaires were distributed to patients in pilot experiment to test whether it was comprehensible and simple to use. The pilot preoperative questionnaire was revised repeatedly by feedback from patients to develop the final preoperative questionnaire. Table 1 showed the descriptions for the ten items in the final preoperative questionnaire. These patients in pilot experiment were not included in our final analysis.

**Table 1:** Descriptions of Potential Adverse Effects

Effects	Descriptions
Anxiety	You feel nervous or worried preoperatively.
Intravenous puncture pain	You feel pain in the upper limb caused by intravenous puncture.
Propofol injection pain	You feel pain in the upper limb caused by the injection of some anesthetics.
Gag on endotracheal tube	You feel difficult to breathe and it is impossible to speak as a breathing tube is in your windpipe when you are awaking.
Shivering	You feel cold and your whole body shakes uncontrollably after surgery.
Sore throat	Your throat is sore and your voice is hoarse after surgery.
Nausea	You want to throw up, but cannot after surgery.
Vomiting	You feel waves of nausea and are throwing up after surgery.
Incisional pain	You feel pain in surgical incision and movement makes it worse.
Normal	You experience no above effects.

Patients older than 18 years with thyroid carcinoma who were scheduled for elective open thyroidectomy under general anesthesia were eligible for our study. They were surveyed before and after surgery. We excluded individuals who had communication problems, were unable to speak or read Chinese, or incapable of understanding the contents of questionnaire. Those who refused to participate were also excluded. We distributed 500 preoperative questionnaires from May 2016 to July 2016 and aimed to collect at least 400 completed questionnaires. All the completed questionnaires were collected anonymously.

The final preoperative questionnaire consisted of two parts: standard demographic items including age, sex, and previous experience of surgery, and a rankings section. In the rankings section, we asked patients to rank the 10 specific items. We used priority ranking scales to determine their preferences. Detailed instructions were given to the included patients to help them understand this ranking technique well. For example, “please rank each of these problems (you might experience) in relation to each other from 1 to 10 (1 = the most undesirable to 10 = the most desirable). Assume that each hypothetical situation occurs with equal frequency and would have the same duration. Each ranking number should be used only once.” One trained investigator (W.W.) who did not participate in patients’ anesthesia was responsible for distributing the questionnaire, explaining and answering any question asked by patients during this process. Moreover, the investigator reminded patients that there was no standard or right answer and they were encouraged to express their own views. Preoperative questionnaires would be considered valid or correct only if the normal outcome was ranked 10 (highest). Thus, we excluded all preoperative questionnaires that were not fully completed or were incorrect in our final analysis.

Our study did not intervene in the anesthetic management of surveyed patients. In our routine anesthesia practice, no premedication was given. A 20-gauge venous cannula was inserted into a forearm vein and standard monitors were placed upon patients’ arrival in the operating room. All patients were induced routinely with midazolam, propofol, opioids, cisatracurium/rocuronium and maintained with propofol or sevoflurane.

Patients who completed the preoperative questionnaires were followed up the first day after surgery. They were asked by the investigator (L.X.W.) whether they could recall any discomfort or pain in the upper limb during anesthetics injection before falling asleep. For those who answered yes, we asked them to grade the severity of pain using a three-point scale (mild, moderate, severe) and compare it with intravenous puncture pain and incisional pain.

### Statistical analysis

Data were presented as frequency and percentage (*N*, %) or mean ± standard deviation (*SD*) when appropriate. Ranking data were analyzed by ANOVA using Student-Newman-Keulsa test. Independent sample *T*-test was used to determine the effect of the demographic data (gender, age and previous experience of surgery) on the ranking data. A *P* value less than 0.05 (two-tailed) was considered statistically significant. All data were analyzed with the SPSS software (Statistical Package for the Social Sciences, version 19.0, SPSS Inc, Chicago, IL, USA).

## Results

A total of 500 preoperative questionnaires were distributed. In this process of data collection, 52 questionnaires were excluded for they were incomplete or incorrect. Finally, 448 valid preoperative questionnaires were analyzed with an efficient rate of 89.6%. Demographic characteristics (gender, age and previous experience of surgery) of the included 448 patients were presented in Table 2. Our study population mainly included middle-aged females. The majority of the patients (86%) were between the ages of 31 and 59 years, 8% under 30 years, and 6% above 60 years. Sixty-three percent of patients were female while 37% were male. Seventeen percent of the study patients had previously experienced a surgical procedure.

**Table 2:** Demographic Characteristics of the Study Patients (*N* = 448)

Characteristics	*N*	%
Age (years)		
≤30	34	8
31–59	388	86
≥60	26	6
Gender		
Male	166	37
Female	282	63
Previous experience of surgery	78	17

Ranking data were shown in Table 3. Incisional pain was the most undesirable experience. As for pain, relief of incisional pain and sore throat was more important than relief of propofol injection pain. However, interindividual variability of patient preferences for the ten events was existed (Table 4). As expected, the hypothetical state “Normal” was ranked as the most desirable event. The demographic data (gender, age and previous experience of surgery) did not significantly affect patients’ ranking data.

**Table 3:** Ranking of Potential Adverse Effects

Potential Adverse Effects	Ranking
Incisional pain	2.53 ± 2.38
Vomiting	4.21 ± 2.50
Gag on endotracheal tube	4.37 ± 2.24
Nausea	4.66 ± 2.23
Sore throat	4.67 ± 2.31
Propofol injection pain	5.65 ± 2.00
Shivering	5.67 ± 2.39
Intravenous puncture pain	6.02 ± 2.39
Anxiety	6.18 ± 3.27
Normal	10.00

Values are presented as means ± *SD. SD* standard deviation

**Table 4:** Number and Percentage of Patients’ Ranking of Potential Adverse Effects

Potential Adverse Effects	Rankings									
	1	2	3	4	5	6	7	8	9	10
Anxiety (*N*)	83	31	15	14	18	25	17	41	204	0
%	18.5	6.9	3.3	3.1	4.0	5.6	3.8	9.2	45.5	0
Intravenous puncture pain (*N*)	20	33	35	38	39	54	42	141	46	0
%	4.5	7.4	7.8	8.5	8.7	12.1	9.4	31.5	10.3	0
Propofol injection pain (*N*)	8	27	44	55	58	63	120	48	25	0
%	1.8	6.0	9.8	12.3	12.9	14.1	26.8	10.7	5.6	0
Gag on endotracheal tube (*N*)	54	58	63	56	71	62	41	26	17	0
%	12.1	12.9	14.1	12.5	15.8	13.8	9.2	5.8	3.8	0
Shivering (*N*)	28	31	36	44	56	64	72	54	63	0
%	6.3	6.9	8.0	9.8	12.5	14.3	16.1	12.1	14.1	0
Sore throat (*N*)	35	63	57	71	61	46	52	37	26	0
%	7.8	14.1	12.7	15.8	13.6	10.3	11.6	8.3	5.8	0
Nausea (*N*)	32	53	71	72	57	60	40	43	20	0
%	7.1	11.8	15.8	16.1	12.7	13.4	8.9	9.6	4.5	0
Vomiting (*N*)	65	86	63	43	48	40	41	35	27	0
%	14.5	19.2	14.1	9.6	10.7	8.9	9.2	7.8	6.0	0
Incisional pain (*N*)	123	66	67	54	42	33	22	22	19	0
%	27.5	14.7	15.0	12.1	9.4	7.4	4.9	4.9	4.2	0
Normal (*N*)	0	0	0	0	0	0	0	0	0	448
%	0	0	0	0	0	0	0	0	0	100

The incidence and intensity of recall of PIP on the first postoperative day was presented in Table 5. The majority (91.5%) of surveyed patients could not recall any discomfort or pain in the upper limb during anesthetics injection before falling asleep. Of those who could recall, 89.5% grade it as mild pain, 7.9% moderate pain, and 2.6% severe pain. Overall, most patients considered PIP as a relatively minor problem. Those who recalled mild PIP felt it was temporary, acceptable, and lighter than intravenous puncture pain. Only one patient in our study recalled severe PIP, which was caused accidentally by taking blood pressure during propofol injection on the ipsilateral upper arm.

**Table 5:** Incidence and Intensity of recall of PIP on the first postoperative day

Recall of PIP	*N*	%
None	410	91.5
Recall	38	8.5
Mild	34	89.5
Moderate	3	7.9
Severe	1	2.6

*PIP* propofol injection pain

## Discussion

Propofol has been widely used in clinical practice. However, pain after injection is one of the most common side effects of this intravenous anesthetic. It has been reported that propofol injection pain (PIP) occurred in 60% of untreated patients [9]. This common but low-morbidity clinical anesthesia problem has been paid enough attention by researchers over the last decades. According to the study published in 2011 [10], 177 trials including more than 25 000 participants have been conducted to find effective interventions to prevent PIP. Moreover, more than 30 published clinical trials were yielded when we searched the online database of Pubmed in recent 5 years using the following term of “propofol injection pain”.

However, is PIP really important to patients? Maybe such pain is considered important just by clinicians but not patients. Besides, as midazolam and propofol are commonly used in anesthesia induction, the amnesic effects of the two drugs may blunt recall of PIP after surgery [11–14]. In our study, the preference assessment tool of priority ranking was used to study patient preferences and determine their opinion on the problem of PIP. Incisional pain was ranked as the most undesirable, followed (in order) by vomiting, gagging on the tracheal tube, nausea, sore throat, propofol injection pain, shivering, intravenous puncture pain, and anxiety. Most patients view propofol injection pain as a relatively minor problem. Under routine general anesthesia in our hospital, the incidence of recall of PIP was low (8.5%) and the majority of those who recalled (89.5%) regarded PIP as mild, temporary, acceptable, and lighter than intravenous puncture pain.

So far, PIP has been adequately studied with plenty of labor forces and financial resources inputted. A great many effective non-pharmacological and pharmacological approaches have been recommended to prevent or alleviate PIP including venous occlusion by a tourniquet [15], alternative propofol formulations [16, 17], changing physical properties of propofol [18, 19] and pretreatment with lidocaine or opioids [20–23]. However, none of these can eliminate PIP completely. Besides, as a practical matter, many above methods are not routinely available and will delay busy operating room schedules. The most pragmatic option for preventing PIP should be the simple effective method that allows clinicians to use routinely available drugs and avoids delay to busy operating room schedules [10]. Thus, lidocaine and opioids, either as a pretreatment or mixed with propofol, are reasonable options as they are routinely administered during induction of anaesthesia and convenient to perform in busy clinical situation [10]. In our hospital, PIP is not adequately managed as lidocaine is not routinely used during induction of anesthesia. Even in this case, though, the incidence of recall of PIP was low and most patients view PIP as a relatively minor, acceptable problem. Maybe we should no longer need to spend too much time, energy and financial resources on the problem of PIP. Although there is interindividual variability in patient preferences, our study showed that avoiding incisional pain, nausea and vomiting still seems to be major concerns of patients. This finding is consistent with previous studies [3, 7]. Thus, maybe priority should be given to the management of postoperative pain, nausea and vomiting and more time and resources should be spent to minimize these distressing clinical problems.

There are some limitations to our study. Firstly, potential selection bias may exist in our study as the sample of patients surveyed was from a single tertiary specialized cancer hospital scheduled for elective open thyroidectomy under general anesthesia. We do not know whether similar results would be obtained in patients undergoing major surgeries or in patients with chronic pain conditions. Patients’ perspectives on PIP and its severity may differ between patient populations. Thus, the finding in our study should be generalized to other patient populations carefully and further investigations into PIP may be warranted. Secondly, our study population mainly included middle-aged and relatively well-educated patients. As cognitive biases may affect the results of survey based study [24], it is unknown if the results represent the general population. Thirdly, we only investigated the ranking of PIP by the patients before surgery. However, it would have been useful to compare the rankings of PIP by the patients before and after surgery. Fourthly, propofol was applied to induction of anesthesia in our study. We do not know whether there is a difference between using propofol for sedation and using it for induction with respect to patient recall. Fifthly, only nine potential side effects were given and patients’ preferences may be changed when other side effects were included. Finally, although priority ranking was a common method to study patients’ preference, the subjective evaluation may not always reflect rational choice.

## Conclusions

In our study, we found that most of patients undergoing elective open thyroidectomy viewed propofol injection pain as a relatively minor problem. The incidence of recall of propofol injection pain was low and the majority of those who recalled regarded it as temporary and acceptable pain, which was lighter than intravenous puncture pain. However, as patients’ perspectives on propofol injection pain and its severity may differ between patient populations, further investigations into propofol injection pain may be warranted.

## Abbreviations

PIP: Propofol injection pain
SD: Standard deviation
